# The Genomic Diversity and Phylogenetic Relationship in the Family *Iridoviridae*

**DOI:** 10.3390/v2071458

**Published:** 2010-07-15

**Authors:** Heather E. Eaton, Brooke A. Ring, Craig R. Brunetti

**Affiliations:** Trent University, Peterborough, ON, K9J 7B8, Canada, E-Mails: heathereaton@trentu.ca (H.E.E.); brookering@trentu.ca (B.A.R.)

**Keywords:** *Iridoviridae*, evolution, repetitive sequences

## Abstract

The *Iridoviridae* family are large viruses (∼120–200 nm) that contain a linear double-stranded DNA genome. The genomic size of *Iridoviridae* family members range from 105,903 bases encoding 97 open reading frames (ORFs) for frog virus 3 to 212,482 bases encoding 211 ORFs for Chilo iridescent virus. The family *Iridoviridae* is currently subdivided into five genera: *Chloriridovirus, Iridovirus, Lymphocystivirus, Megalocytivirus,* and *Ranavirus*. Iridoviruses have been found to infect invertebrates and poikilothermic vertebrates, including amphibians, reptiles, and fish. With such a diverse array of hosts, there is great diversity in gene content between different genera. To understand the origin of iridoviruses, we explored the phylogenetic relationship between individual iridoviruses and defined the core-set of genes shared by all members of the family. In order to further explore the evolutionary relationship between the *Iridoviridae* family repetitive sequences were identified and compared. Each genome was found to contain a set of unique repetitive sequences that could be used in future virus identification. Repeats common to more than one virus were also identified and changes in copy number between these repeats may provide a simple method to differentiate between very closely related virus strains. The results of this paper will be useful in identifying new iridoviruses and determining their relationship to other members of the family.

## 1. Introduction

Iridoviruses are large double stranded DNA viruses (∼120 – 200 nm) that replicate in the cytoplasm of infected cells. They are characterized by a distinct icosahedral capsid and range in size from 102 to 212 kbp [1,2]. Iridoviruses are subdivided into five genera that infect a wide range of vertebrate (*Ranavirus, Megalocytivirus, Lymphocystivirus*) and invertebrate (*Iridovirus, Chloriridovirus*) hosts [3]. Specifically, iridoviruses have been found to infect fish, amphibians, reptiles, crustaceans, molluscs, and insects [4]. Following attachment and entry of iridoviruses via receptor-mediated endocytosis, virus particles are uncoated and translocated to the nucleus where the first stage of DNA replication occurs via a virus-encoded DNA polymerase [5,6]. Viral DNA then exits the nucleus to the cytoplasm for the second stage of DNA replication where the formation of DNA concatemers occurs [5]. Iridoviruses are unique among eukaryotic virus genomes because they are described as both circularly permuted and terminally redundant [7–9].

Clinical manifestations of iridovirus infections vary greatly between species and can range from an absence in observable symptoms to death [10–14]. The high morbidity and mortality associated with some iridovirus members has demonstrated their high ecological and economical significance, specifically in aquatic organisms [15–19]. With the continuing isolation of new iridovirus-like viruses from a variety of species worldwide it is imperative to gain a greater understanding of iridovirus pathogenesis.

Fifteen iridoviruses have currently been sequenced including at least one from each genus. They include frog virus 3 (FV3; *Ranavirus*) [20], soft-shelled turtle iridovirus (STIV; *Ranavirus*) [21], tiger frog virus (TFV; *Ranavirus*) [1], epizootic haematopoietic necrosis virus (EHNV; *Ranavirus*) [22], *Ambystoma tigrinum* virus (ATV; *Ranavirus*) [23], grouper iridovirus (GIV; *Ranavirus*) [24], Singapore grouper iridovirus (SGIV; *Ranavirus*) [25], lymphocystis disease virus 1 (LCDV-1; *Lymphocystivirus*) [26], lymphocystis disease virus China (LCDV-C; *Lymphocystivirus*) [27], infectious spleen and kidney necrosis virus (ISKNV; *Megalocytivirus*) [28], rock bream iridovirus (RBIV; *Megalocytivirus*) [29], red sea bream iridovirus (RSIV; *Megalocytivirus* [30], orange-spotted grouper iridovirus (OSGIV; *Megalocytivirus*) [31], invertebrate iridescent virus 6/Chilo iridescent virus (IIV-6/CIV; *Iridovirus*) [2], and invertebrate iridescent virus 3/mosquito iridescent virus (IIV-3/MIV; *Chloriridovirus*) [32]. These 15 sequenced genomes represent iridoviruses isolated from a variety of hosts including fish, amphibians, reptiles, and insects. The diverse host range of iridoviruses is reflected in the diverse gene content found between iridoviruses of different genera.

Little is currently known about the molecular biology of this family of viruses and much about the evolutionary biology of iridoviruses still contains many unanswered questions. Despite many recent advances in molecular phylogenetics, there is much to learn about the relationship of iridoviruses within the family itself. In order to gain a greater understanding of iridovirus evolutionary history, we will use previously identified iridovirus core genes and repetitive DNA sequences to explore the evolutionary links between iridoviruses. An increased knowledge about the evolutionary biology of iridoviruses may lead to a better understanding of the functional biology of these viruses, specifically in the understanding of iridovirus pathogenesis.

## 2. Results and Discussion

### 2.1. Phylogenetic analysis

Whether representing a single gene or a consensus, the *Iridoviridae* family genera branching order is often inconsistent between genomic papers [1,21,23,29,31,33]. The discrepancies between papers might be a result of different sequence alignment methods or comparison of insufficient data sets. In order to clarify the evolutionary relationships of the family *Iridovirida*e, a phylogenetic analysis was constructed using sequence alignments.

**Figure 1:**
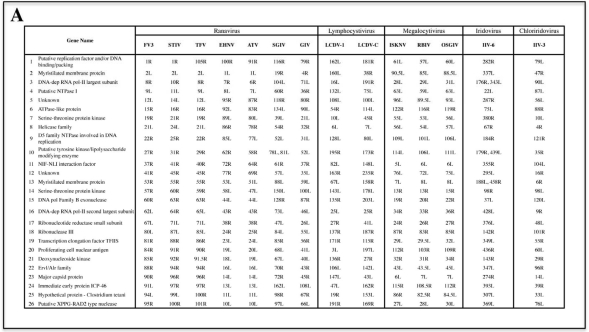

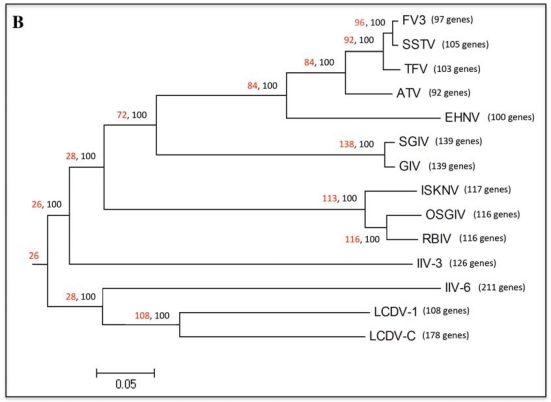
The phylogenic relationship of 14 iridoviruses as identified by comparison of 26 conserved iridovirus genes. **(A)** ORF’s of 26 conserved iridovirus genes were recorded from 14 iridoviruses. **(B)** The nucleotide sequence of the conserved genes were aligned and fused to create a neighbor-joining consensus tree. The numerical values adjacent to the branching nodes indicate bootstrap values (black) and the number of genes in common between genomes (red). This tree was constructed in MEGA4.1 using the p-distance model at 500 replicates.

The open reading frames of 26 conserved iridovirus genes shared by 14 iridoviruses (representing all five genera; Figure 1A) were aligned using ClustalW in BioEdit 7.0.5. The sequence for the RSIV genome is not available and was therefore not included in the analysis. The alignments were then transferred to MEGA4.1 and fused together to create a consensus tree (Figure 1B). The consensus tree is a phylogenetic branching diagram of the *Iridoviridae* family that shows the evolutionary relationships between 14 iridovirus species (Figure 1B). The nodes of the tree demonstrate shared ancestry, and the length of the branches represent an estimation in time, although they can also reflect evolutionary pressures. The tree shows one main common ancestor, which shares 26 conserved genes with modern *Iridoviridae* (Figure 1B). The tree divides into two branches, the first branch consists of the *Iridovirus* and *Lymphocystivirus* genera and the second branch consists of the *Chloriridovirus*, *Megalocytivirus,* and *Ranavirus* genera (Figure 1B). Jakob *et al.* [2] sequenced the IIV-6 genome and found through comparison of 10 core gene products that the *Iridovirus* genus was most closely related to the *Lymphocystivirus* genus, which is consistent with the results of our study [2,27]. Many other studies have argued that the *Iridovirus* genus is more closely related to the *Chloriridovirus* genus however, this may be a result of insufficient data sets which do not include all genera, insufficient numbers of core genes used in the analysis, or different alignment methods [21,28,32,33]. Individual phylogenies showed that the *Iridovirus* genus clustered closer to the *Lymphocystivirus* genus then the *Chloriridovirus* genus in 22 out of the 26 core genes (data not shown). Another phylogeny based on the aligned genomes of IIV-6, IIV-3, LCDV-1 and LCDV-C also clustered the *Iridovirus* genus closer to the *Lymphocystivirus* genus then the *Chloriridovirus* genus (data not shown).

The *Megalocytivirus* genus is composed of very closely related sister taxa whose genomes differ only by 2.3%. The placement of the *Megalocytivirus* genus between the *Chloriridovirus* and *Ranavirus* genera has been previously observed [32]. It should be noted that the *Megalocytivirus* genus is more often observed to cluster with the *Lymphocystivirus* and *Ranavirus* genera, however, this outcome occurs in data sets that do not include the *Chloriridovirus* genus [21,23,28,31]. The relatedness of the *Megalocytivirus* and *Ranavirus* genera has been well documented in previous phylogenetic analysis between genomic papers [21,27,32]. Species within the *Ranavirus* genus are generally very closely related, however, SGIV and GIV are considered outliers as their genomes differ in sequence identity by approximately 30% from the genomes of FV3, STIV, TFV, ATV and EHNV, while the sequence identity within other *Ranavirus* genomes (FV3, STIV, TFV, ATV and EHNV) differ by less than approximately 10% [23,25,34]. The branching order of the individual viruses within the *Ranavirus* genus is consistent with many genomic papers [20,21,27,31,33].

### 2.2. G/C content

**Figure 2:**
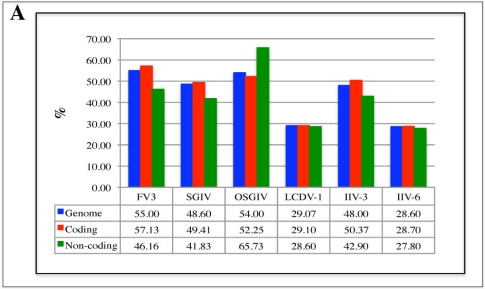

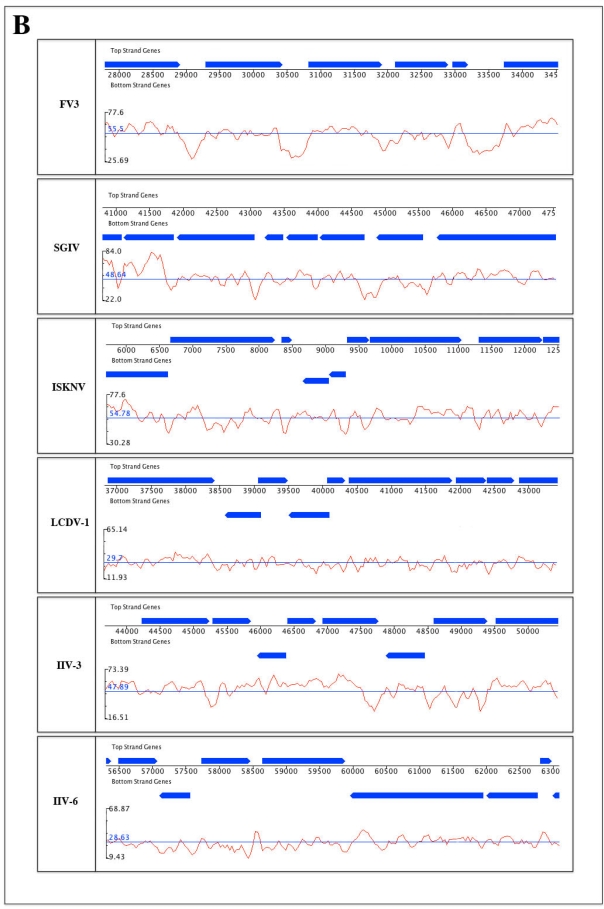
Different iridoviruses exhibit different trends in G/C content of coding *versus* non-coding regions. G/C content of coding regions was determined using the VGO software. The length and G/C content of the coding regions and overall length and G/C content was used to determine the G/C content of the non-coding regions. **(A)** Overall G/C content is shown in blue, coding region G/C content is shown in red, and non-coding G/C content is shown in green. **(B)** Images for one representative virus from each genus are displayed showing changes in G/C content (red line) in selected coding (blue bars) and non-coding regions of the genome. Average genome G/C content is displayed in blue.

G/C content of specific genes can provide information about gene origins (*i.e.*, host-derived genes). Comparison of iridovirus gene G/C content with host species G/C content did not show evidence of apparent host gene transfer, but we did find evidence of specific trends in G/C content throughout iridovirus genomes. We used the VGO software to identify the G/C content of each coding and noncoding region of the sequenced iridoviruses (excluding STIV, EHNV, and RSIV which are not included in the VGO database) [35]. For some genera (*Ranaviruses, Chloriridovirus*), coding regions were found to exhibit higher G/C content than both the average G/C content of the virus and the G/C content of non-coding regions (Figure 2A). Figure 2B clearly shows that for coding regions of FV3, SGIV, and IIV-3, G/C content fluctuates around or above the average G/C content for the virus. G/C content in non-coding regions dips dramatically below the average G/C content (Figure 2B). This trend is most likely due to the fact that promoters are often A/T rich and therefore regions upstream of coding regions will contain a higher A/T content with respect to other locations in the genome. Specifically, large DNA virus promoters (*Poxviridae, Iridoviridae, Asfarviridae* families) have been described as A/T rich [36–39]. In contrast, viruses of the *Megalocytivirus* genus show the opposite trend of the *Ranavirus* and *Chloriridovirus* genera (Figure 2A and 2B). G/C content of non-coding regions is higher than both overall genome and coding region G/C content. Furthermore, some genera (*Lymphocystitvirus, Iridovirus*) do not show any differences in G/C content throughout any part of their genome. This is most likely a result of a much lower overall G/C content than the *Ranavirus* and *Chloriridovirus* genera. These results demonstrate that variations in G/C content between non-coding and coding regions could provide an alternative method in annotating genomes, specifically in the *Ranavirus* and *Chloriridovirus* genera. The trends seen in G/C content between coding and non-coding regions may also help to classify new viruses or virus strains into their appropriate genus.

### 2.3. Iridoviridae repetitive sequences

While comparison of conserved genes and phylogenetics are valuable tools for exploring the evolution of viruses, the molecular differences between genomes is critical for understanding their evolutionary relationships. Repetitive sequences are key when assessing gene and genome diversity and provide a method for examining the evolution of viruses [40]. Simple sequence repeats are often widely abundant in genomes, and have been identified not only in a wide variety of eukaryotic and prokaryotic genomes, but also in several virus families. Specifically, dsDNA viruses including poxviruses, herpesviruses, baculoviruses, and adenoviruses have been found to contain extensive repeat sequences within their genomes [41–45]. Abundant numbers of repetitive sequences are also found throughout iridoviruses genomes [2,20,21,23–28,31,32], however no comparative approach has been taken to analyze the repeats between all iridoviruses. We are interested in determining the distribution and abundance of repeat elements throughout the sequenced genomes of the family *Iridoviridae*.

A DNA tandem repeat finder identified tandem repeats found within all available sequenced iridovirus genomes [46]. Repeats between 1–6 nucleotides in length are referred to as microsatellites and were found only rarely in iridoviruses (Table 1, Supplementary Tables 1 – 7). Repeats containing a minimum of 6–12 nucleotides are referred to as minisatellites. This type of tandem repeat was found extensively through all iridovirus genomes (Table 1, Supplementary Tables 1 – 7). The copy number of the repeats also varied within and between genomes (Table 1, Supplementary Tables 1 – 7). Variability in repetitive sequences can occur through many mechanisms including recombination and slipped strand mispairing [47]. Inefficient polymerase activity and poor repair mechanisms during DNA replication can result in alterations within a repeat sequence [48,49].

**Figure 3:**
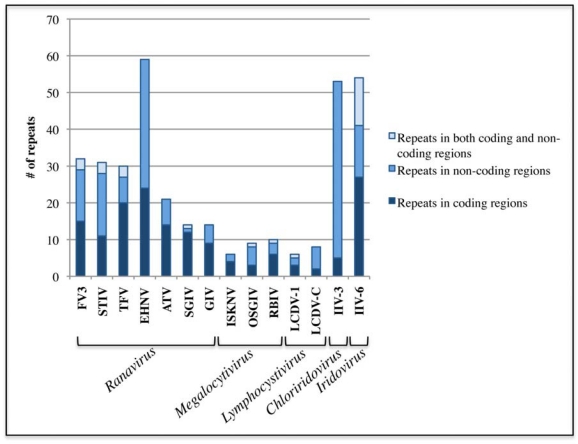
Repeats in the family *Iridoviridae* vary in number and dispersion throughout the genomes. Tandem repeats were identified using a tandem repeat finder [46]. For each sequenced iridovirus, the number of tandem repeats found within coding regions are shown in dark blue, repeats found within non-coding regions are shown in blue, and repeats that transverse between both coding and non-coding regions are shown in light blue. The height of the bar represents the total number of repeats found in the virus.

Repetitive sequences can be grouped into categories based on several factors, one of those being their respective locations to coding regions in a genome. The repetitive sequences analyzed in the sequenced iridovirus genomes were widely dispersed and were located in both non-coding and coding regions (Figure 3). Furthermore, the majority of genomes contained individual repeats that transverse between both coding and non-coding regions (Figure 3). Repetitive sequences were also identified to occur within the core iridovirus genes of many viruses. While the current function of these repeats is unknown, simple sequence repeats have been found to influence gene regulation, transcription, and protein function (reviewed in [50]). Repetitive sequences can act as structural elements as well as binding sites for proteins [50]. This modulation in gene expression may be the result of changes in repeat number that alter the physical integrity of DNA domains as coding regions become modified or disrupted. Specifically, simple sequence repeat involvement in the modulation of gene expression has been recently identified in a number of microorganisms, including viruses [51–55]. In addition, changes in repeat number can cause quantitative changes in gene expression and function, which may lead to variation between similar viruses. For example, host range and pathogenicity could be affected by differences in repeat number between viruses. As more and more sequenced genomes become available it provides an opportunity to study the involvement of these repeat sequences and their effect on gene function and pathogenicity.

The number of total genomic repeats varied dramatically between iridoviruses and the number of repeats that were similar between viruses varied greatly (Figure 3). Large numbers of repeats were found in the *Ranavirus*, *Iridovirus*, and *Chloriridovirus* genera, while the *Lymphocystivirus* and *Megalocytivirus* genera exhibit relatively fewer repeats (Figure 3, Table 1, Supplementary Tables 1 – 7). Not only were fewer repeats present in the genomes, but also the copy numbers of the repetitive sequences were fewer than in other genera.

No repetitive sequences were shared between all iridoviruses or even between all members of one genus. In the *Lymphocystivirus* genus, no repeats were shared between LCDV-1 and LCDV-C, which is expected because the whole genome sequence identity between these two viruses is extremely low (14%; Supplementary Table 4). No repeats were shared between all members of the *Megalocytivirus* genus. However, all the repeats found within RBIV are shared with OSGIV, both in terms of sequence identity and copy number. This suggests that these viruses are extremely closely related and most likely strains of the same virus. ISKNV does not share any repetitive sequences in common with RBIV and OSGIV even though the sequence identity between ISKNV and RBIV or OSGIV is relatively high (97%; Supplementary Table 3). The relationship between viruses of the *Megalocytivirus* genus shown in Figure 1B is clearly supported using repetitive sequence data. While IIV-3 and IIV-6 contain many tandem repeats with varying sizes and copy numbers, the repeats are not similar to each other or to other iridoviruses (Supplementary Table 5, 6).

### 2.4. Ranavirus repetitive sequences

Several shared repeats were found within the subset of ranaviruses that includes FV3, STIV, TFV, EHNV, and ATV (Table 1). These viruses have relatively high sequence identity between one another (>91%) and are similar in terms of size and G/C content. While no repeat was shared between all of these viruses, some repeats were found to be identical between 2 to 4 of these viruses (Table 1). A single repeat was found to be common between FV3, STIV, TFV, and EHNV (Table 1: FV3 indices - 70421) while another single repeat was found to be common between FV3, STIV, EHNV, and ATV (Table 1: FV3 indices - 6616). Although these viruses have relatively high sequence identity, the commonality between their repetitive sequences was found to be low. Only 3 repeats were found to be in common between FV3, STIV, and TFV (Table 1; FV3 indices – 70421, 22509, 54981)), which is surprising considering the sequence identity between these three viruses is greater than 97%. Overall, 6 repeats from FV3 (FV3 indices – 70421, 22509, 54981, 100057, 51310, 18613), 7 from STIV (STIV indices – 70191, 22236, 54757, 39783, 38839, 39061, 28871), 2 from EHNV (EHNV indices – 54319, 88097), and 1 from ATV (ATV indices – 60741) were found to be in common with TFV. Furthermore, only 2 repeats from FV3 (FV3 indices – 6616), 3 from STIV (STIV indices – 6390, 38839, 800), 1 from TFV (TFV indices - 37464, and 3 from EHNV (EHNV indices – 5374, 125666, 25889) were found to be similar to ATV. The lack of conserved repeats between ATV and other viruses of this subset of ranaviruses is not surprising due to the fact that ATV has a much lower sequence identity with FV3, STIV, EHNV, and TFV then they do with each other. While there are very few conserved repeats between ATV and FV3, STIV, TFV, and EHNV, there are no repeats in common between ATV and the second subset of ranaviruses (GIV, SGIV). This confirms the evolutionary relationship between the *Ranavirus* genus found in Figure 1B. It suggests that ATV is more closely related to FV3, STIV, TFV, and EHNV, than with GIV and SGIV, but that FV3, STIV, and TFV are more closely related to each other then with ATV. EHNV, a ranaviruses with highest sequence identity to ATV, contains a substantial number of repeats within its genome compared to other ranaviruses. Interestingly, most of these repeats are unique only to EHNV, even though EHNV shares relatively high sequence identity to the other ranaviruses (FV3, STIV, TFV, ATV; >97%).

**Figure 4:**
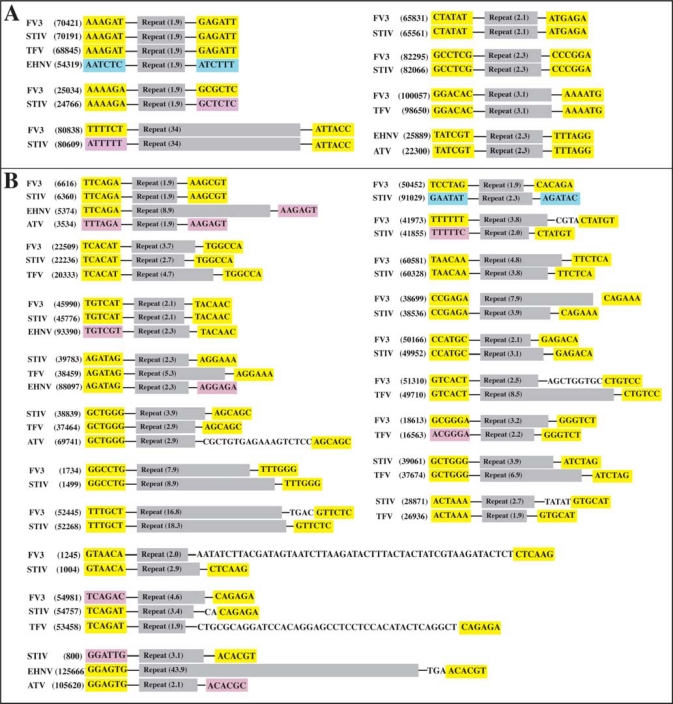
Repeat flanking regions are highly conserved. Regions flanking the repeats common to 2 or more viruses from the first subset of *Ranaviruses* (FV3, STIV, TFV, EHNV, ATV) are shown. Flanking regions exhibiting perfect conservation are shown in yellow. Flanking regions that show high conservation but with one nucleotide change are shown in pink. Flanking regions demonstrating no conservation are shown in blue. Repeats are displayed in gray and the copy number of each repeat is displayed in black. **(A)** Repetitive sequences that display identical copy numbers and **(B)** repetitive sequences that display changes in copy numbers are shown.

Although FV3 and STIV share 99% sequence identity, they share just over 50% of repeats in common with each other (Table 1). This suggests that much of the difference between these two viruses lies within the repetitive regions. While the match identity between repeats found in these two viruses is high, a large change in copy number between repeats is evident (Table 1, Figure 4). Changes in copy number between matching repeats may provide a simple method to differentiate between what is most likely strains of the same virus. Furthermore, a dinucleotide repeat (microsatellite) was identified to be common only between FV3 and STIV. This specific microsatellite may be exploited as a rapid method to identify FV3 or viruses with extremely high similarity to FV3.

While GIV and SGIV share no repeats in common with the first subset of ranaviruses, they do share several repeats in common with each other (Supplementary Table 2). Similar to the sequence identity between FV3 and STIV, GIV and SGIV are 99% similar but share only 50% of repeats in common. This again suggests that the differences between these two viruses lies within the repetitive sequence regions. While the repeats that are common between SGIV and GIV share high sequence identity with each other, they do exhibit large changes in copy numbers.

Our analysis of repetitive regions within sequenced iridoviruses has successfully identified both unique and similar repeats. Identical repeats that exhibit differing copy numbers can be used in conjunction with unique repeats to quickly and effectively identify iridoviruses. This has previously been tested by Jancovich *et al.* [16], in which a 16 base pair preset in ATV was used to help distinguish ATV isolates from Arizona, Utah, Colorado, and Canada. This technique may specifically apply to the identification of several isolates of FV3-like species listed by Hyatt *et al.* [56]. In the past decade there has been a significant increase in the number of iridoviruses found in vertebrates, many of which have yet to be properly classified [1,2,4,15,21,23,56]. The use of repetitive sequences are predicted to be sufficient in determining otherwise undetermined isolates of FV3-like viruses that infect reptiles, amphibians and fish. Due to their high mutation rate in copy numbers, repetitive sequences are considered ideal genetic markers and may provide an efficient method to distinguish between highly similar virus strains and further clarify the evolutionary link between viruses of this family.

### 2.5. Repetitive sequence flanking regions

Repetitive sequences may change in orders of magnitude faster than non-repetitive regions of the genome and are prone to deletions and duplications. In order to determine whether the regions flanking the repetitive sequences mutate slower than the repetitive sequences themselves, we compared the genetic sequence of the regions flanking the repeats that were common to more than one virus. If the repetitive regions mutate faster than other locations in the genome, then the flanking regions should exhibit fewer changes when compared to each other, even when the copy number of the repeat changes. Figure 4 shows the flanking regions (6 nucleotides on either side of the repeat) of each identified tandem repeat common between 2 or more viruses of the first subset of *Ranaviruses* (FV3, STIV, TFV, EHNV, ATV). The majority of flanking regions surrounding a single repeat were highly conserved between viruses. For repeats in which the copy number did not change between viruses, the flanking regions exhibited extremely high conservation with only a few small nucleotide changes being evident (Figure 4A). For repetitive sequences that exhibited changes in copy numbers, there still remained a high level of conservation in the flanking regions with only some small nucleotide changes evident (Figure 4B). Copy numbers that do not form an even number (*i.e.*, 7.9) indicate that the final repeat is not complete. Interestingly, when comparing some viruses in which the copy number of the repeat differed, there were a small number of poorly conserved nucleotides present prior to the conserved flanking region. This occurred only at flanking regions to the right of the repeat and this extra nucleotide sequence generally contained partial sequences from the actual repeat. This suggests that this region represents a former repeat copy that has been altered due to recent insertions, deletions, or recombination. Regions flanking repetitive sequences within the second subset of *Ranaviruses* (GIV, SGIV) and the genus *Megalocytivirus* showed almost perfect conservation (Supplementary Figure 1).

The flanking regions exhibited high levels of sequence conservation as compared to the repetitive sequences. Due to the fact that the repetitive sequences exhibit changes in copy numbers between identical repeats, they create polymorphisms that can easily be detected by PCR using flanking primers.

## 3. Experimental Section

*Phylogenetic analysis:* Nucleotide sequences for each gene were obtained for the 14 sequenced iridovirus genomes from the NCBI website [57]. Genes were blasted by BLASTn against each other to find the optimal homology and the ORFs were determined and recorded for the 26 conserved iridovirus genes. The nucleotide sequences of each gene were transferred to the program BioEdit 7.0.5 and aligned individually through ClustalW (multiple alignment). All gaps were striped to ensuring no false divergence conclusions. Each gene alignment was fused when transferred to MEGA4.1 and a neighbor-joining bootstrap consensus trees was constructed using a p-distance model at 500 replicates.

*Orthologous gene analysis:* Orthologous genes were identified using the Viral Orthologous Cluster (VOC) software [58,59]. Genome sequences were obtained from the VOC database. Orthologous genes identified by the VOC software were confirmed by a BLAST search.

*G/C content analysis:* G/C content analysis was completed using the Viral Genome Organizer (VGO) software [35,59]. The sequence for each genome was obtained from the VGO database. The software identified the G/C content of coding regions and this data was used to determine the G/C content of non-coding regions.

*Repeat analysis:* DNA tandem repeats were identified using a DNA tandem repeat finder [46]. The alignment parameters were 2, 7, 7 for match, mismatch, indel respectively. The minimum alignment score was 50 and the maximum period size was 500. Repetitive sequences with less than 95% match identity are provided but were not included in the analysis (Supplementary Table 7). Genomic sequences were obtained from the NCBI and downloaded in Fasta format [57]. The accession numbers are as follows: FV3 (AY548484), STIV (EU627010), TFV (AF389451), EHNV (FJ433873), ATV (AY150217), GIV (AY666015), SGIV (AY521625), LCDV-1 (L63545), LCDV-C (AY380026), ISKNV (AF371960), RBIV (AY532606), OSGIV (AY894343), IIV-6/CIV (AF303741), IIV-3/MIV (DQ643392). The sequence for RSIV is unavailable and was not included in analysis.

## 4. Conclusions

By aligning 26 core genes and establishing the shared genes between the complete genomes of 14 iridoviruses, the evolutionary phylogeny of each genera of the *Iridoviridae* family was determined. The phylogeny showed that the tree shared one common ancestor, which then split into two groups consisting of the *Iridovirus* and *Lymphocystivirus* genera and the *Chloriridovirus*, *Megalocytivirus,* and *Ranavirus* genera. The repetitive sequences confirm this phylogenetic relationship. They also demonstrate that much of the difference between viruses with high sequence identity lies within the repeat regions. Repeats both unique to each virus and repeats found within more than one virus were identified and provide a simple and effective method to explore the evolutionary relationship between this family of viruses. The repetitive sequences identified in this paper can be used to compare to newly isolated virus strains in order to find the relationship of that virus to known iridoviruses. Our analysis of the flanking regions suggests that these regions can be used to create primers required to detect changes in copy number between repeats shared between viruses. Once created, these primers will enable a fast and simple method to uniquely identify a specific virus or differentiate between closely related iridoviruses.

## Supplementary Materials

















## Figures and Tables

**Table 1:**
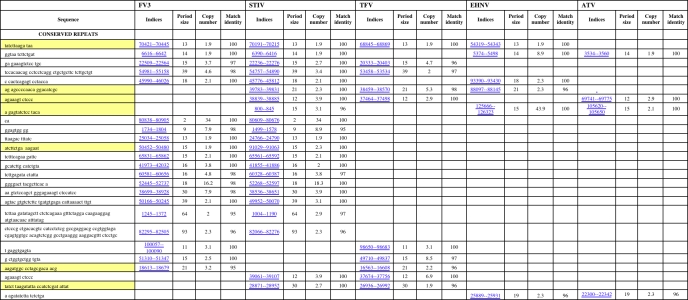
Conserved repeats found in FV3, STIV, TFV, EHNV, and ATV. Repetitive sequences highlighted in yellow represent a repeat that differs in one nucleotide between viruses.
