# Supplementary material for: The Genomic Diversity and Phylogenetic Relationship in the Family Iridoviridae
Source: Viruses. 2010 Jul 15;2(7):1458–75. doi: 10.3390/v2071458 (PMC3185713; doi:10.3390/v2071458)
Supplement: Supplementary file 1 [file viruses-02-01458-s001.pdf]

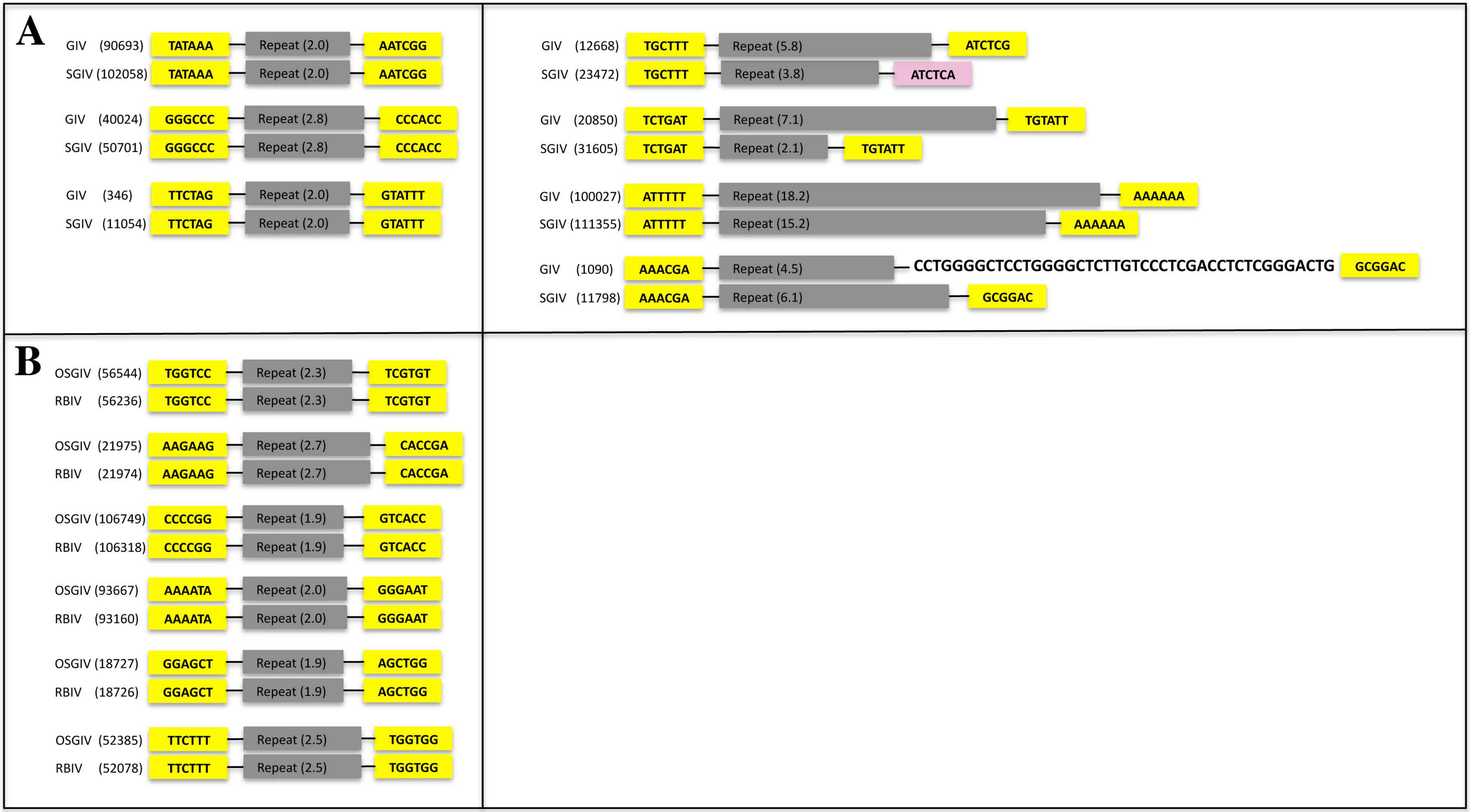

Supplementary figure 1: Flanking regions of *Ranavirus* and *Megalocytivirus* repeats are highly conserved
